# Autistic phenomenology: past, present, and potential future

**DOI:** 10.3389/fpsyg.2023.1287209

**Published:** 2023-12-27

**Authors:** Jonathan Green, Nicola Shaughnessy

**Affiliations:** ^1^Division of Psychology and Mental Health, University of Manchester, and Royal Manchester Children's Hospital, Manchester, United Kingdom; ^2^School of Arts, University of Kent, Canterbury, United Kingdom

**Keywords:** autism, autistic phenomenology, phenomenology, psychopathology, nosology, auto-ethnography, lived experience, citizen science

## Abstract

We are now at a transition point in autism conceptualisation, science, and clinical practise, where phenomenology could play a key role. This paper takes a broad view of the history of phenomenological perspectives on the autism concept and how this has evolved over time, including contemporaneous theory and methods. Early inquiry from a clinical perspective within the tradition of classical continental phenomenology, linked closely to the consideration of schizophrenia, is contrasted with emerging observations of child development and a period in the second half of the twentieth century of scientific inquiry into a behavioural autistic phenotype where there was little or no phenomenological aspect; a phenotype that has determined the recent scientific and clinical conceptualisation of autism within current nosology. We then mark a more recent reawakening of interdisciplinary interest in subjective experience and phenomenological inquiry, which itself coincides with the increasing prominence and salience of the neurodiversity movement, autistic advocacy, and critical autism studies. We review this emerging phenomenological work alongside a contemporaneous clinical phenomenology perspective and representations of autistic experience from within the extensive literature (including life writing) from autistic people themselves; all perspectives that we argue need now be brought into juxtaposition and dialogue as the field moves forward. We argue from this for a future which could build on such accounts at a greater scale, working toward a more co-constructed, systematic, representative, and empirical autistic phenomenology, which would include citizen and participatory science approaches. Success in this would not only mean that autistic experience and subjectivity would be re-integrated back into a shared understanding of the autism concept, but we also argue that there could be the eventual goal of an enhanced descriptive nosology, in which key subjective and phenomenological experiences, discriminating for autism, could be identified alongside current behavioural and developmental descriptors. Such progress could have major benefits, including increased mutual empathy and common language between professionals and the autistic community, the provision of crucial new foci for research through aspects of autistic experience previously neglected, and potential new supportive innovations for healthcare and education. We outline a programme and methodological considerations to this end.

## Introduction

The concept of autism has had a central role in cultural and clinical thinking throughout the twentieth and twenty-first centuries but has lacked a developed and representative phenomenology. Accounting for this will lead us to the heart of many current debates and dilemmas in this field. As a clinical concept, autism has historically been attended to and interpreted by clinicians and philosophers and, until recently, has very little representation of autistic voices themselves. This does not reflect the evolving preoccupations and understanding of the current time. In this paper, we argue that autism conceptualisation and clinical practise have reached a pivotal point, making a representative and systematic phenomenology approach possible. We offer suggestions as to how we can progress beyond this situation and the importance and value of so doing.^1^

## The autistic concept in classical phenomenology

Clinical descriptions are interested in both the *content* and *form* of another's experience to reach toward the empathic understanding of an individual, but also toward more general theories of mental function. The first conceptualisation of autism as a clinical concept is attributed to Bleuler from 1911 as part of early efforts to understand and theorise psychotic states in schizophrenia. He took the name from its Greek root to refer to an “elaborated self-ness” marked by an intense fantasy life linked with withdrawal or disconnection from a shared social world with others. The term was taken up by later psychoanalytic and developmental writers as they refracted evolving contemporary theories of mental life, a history that continues (Evans, 2013). It became associated with psychoanalytic concepts of “primary narcissism”, and, as later amplified by Klein, an “autistic stage” became something conceptualised as part of all infants' early development before their emerging relational engagement with the world. Psychiatry linked to an evolving existential tradition focused more on the state of autistic being. Minkowski, for instance, emphasised the (autistic) “loss of vital contact with reality” and lack of interpersonal awareness or attunement to the environment (Parnas and Bovet, 1991). For the existential psychiatrist Ludwig Binswanger, autism was understood as a radical loss of the central Heideggerian concept of *Dasein*—an authentic and freely embedded “being-in-the-world”. It was this “loss of self and the world” (Needleman, 1968, p. 388) that characterised the autistic state rather than an overly internally directed drive as understood from analytic theory. Binswanger's essentially relational understanding of being focused on the quality of interpersonal experience with autistic people—as a lack of their shared “common sense” or intersubjective horizon with others—which later came to be used as an intuitive criterion for clinicians in what was called atmospheric diagnosis (i.e., based on an experience of being with the other). Autistic experience is here characterised as something fundamentally differing and in parallel to a normatively shared world; for Jaspers, a differing world tending to be essentially “not knowable” (Erklären), while for Binswanger, fundamentally different but approachable with an effort of relational imagination (Hoffmann and Knorr, 2018).

What can we take from this early tradition? There remains a question as to whether these early accounts were really of individuals we would now understand as autistic, but the articulated autism concept, in its location at the junction of selfhood and social life, encapsulated and became a focus-case for many general issues which preoccupied, and still preoccupy, the humanities and the human sciences. Autism raises questions around interpersonal being-in-the-world and inter-subjectivity that remain relevant and central to our tradition. These early clinical formulations of Bleuler's elaborated *self-ness* and Binswanger's sense of an autistic loss of absorption (he uses the term *gestimmtheit* or attunement) within a shared world, along with an apparent “unknowability” from others, receive vivid commentary (as well as some key challenges) in contemporary experiential accounts from autistic people themselves, as we will see below. Some of this classic clinical phenomenological writing has also continued into the present, but in the context of schizophrenia rather than autism itself, as a primary disruption of *self-ness* or *ipseity* and existential presentness as what are still termed “autistic” states within psychosis (for instance, Sass and Parnas, 2003).

From early on, autism was also a focus within an emerging child developmental science (methodologies directly involving children rather than inference from adult recollection). Piaget (1929) used observation and phenomenological-type interviews with a small group of children, particularly his own daughters, in conceptualising a sequence of emerging developmental engagement with reality from what he considered (echoing analytic stage theory) normative autistic beginnings to fully-fledged adult cognitive and moral engagement with the world. Gesell and Armatruda (1945) used more systematic empirical observation (including creative early photography and video) on larger samples in early child development to describe an “embryology of behaviour”, understood as an unfolding process analogous with foetal embryology. Rather than a normative stage, they inferred a developmental immaturity account of early psychosis and autistic behaviours; indeed, this work was antecedent to later developmental science demonstrating, in contrast to a normative autistic phase, the exquisite environmental responsiveness and connectedness of all babies. Normative child development milestones in language, motor, cognitive or social skills came to be codified based on significant divergence from population means. Clinicians such as Sukhareva and later Asperger published descriptive accounts of recognisably autistic teenagers and adults but less focussed on phenomenology, against a background of described intellectual disability and personality development. Sukhareva is notable particularly for her early clinical accounts from 1926, including with girls (Simmonds and Sukhareva, 2020; Sher and Gibson, 2021). The classic early clinical accounts of “infantile autism” (Kanner, 1943) also grew from this framework of observed early child development but mixed still with psychoanalytic stage theory.

## A “behavioural turn” and loss of subjectivity

An emerging empirical psychiatry in the 1960s and 70s took strategic decisions to focus exclusively on precise and repeatable observations of autistic behaviour, particularly within early childhood (in the tradition initiated by Gesell), actively excluding the subjective or experienced self from consideration. In this way, it radically re-cast the autism concept as an intended atheoretical and empirical “behavioural phenotype” within epidemiological enquiry (Evans, 2013). This was partly to make the science easier but also to make decisive distance from ongoing analytically informed debates on the subjective internal world nature and causes of autism. This “behavioural turn” did meet resistance. A prominent clinical child psychiatrist wrote that it was “…impossible to use purely behavioural criteria if we were to convey what we all felt to be the heart of the matter—namely the presence of an impaired capacity for human relationships” (Creak, 1961). Later, the eminent US academic Leon Eisenberg worried about a move toward “mindless psychiatry” (Eisenberg, 1986). Nevertheless, this behavioural turn can be credited with great scientific success in its own terms. Efforts toward a shared behavioural phenotypic account of autism, plus identifying it as an early childhood condition (with the associated views that internal mental states would be impossible to access), enabled a reliable psychometrically valid and shared empirical description to be established that facilitated much international science for 50 years. In turn, this behavioural focus was followed, by nosological and clinical language within the formulations of the International Classification of Diseases and Diagnostic and Statistical Manual of Mental Disorders. The autism concept was reframed and distinguished from both schizophrenia and phenomenology (Barale et al., 2019); however, any newer autistic phenomenology reflecting this emerging developmental understanding was missing. Evans (2013) describes a key conceptual transformation in this, from understanding autistic states as an elaboration of recursive fantasy substituting for lack of contact with reality to the idea of autism as a “lack of affective contact” or a “social impairment”. Linked to it was the rise of a cognitive tradition of research focused on deficits in the “theory of mind” and reflexivity. This focus, in the establishment of the idea that autistic people were unable to be reflective or accurate witnesses to their own experience, became another reason for a first-person autistic phenomenology being thought impossible and a growing literature of subjective accounts of autism largely sidelined from scientific consideration.

## A shifting paradigm and re-emergence of phenomenology

Much has changed in recent years to challenge this view. Firstly, the cognitive focus in autism has rather run its course; the differences in the “theory of mind” ended up being non-specific to autism and confounded by expressive language difficulties and IQ variation. Secondly, there has been the rise into prominence of a neurodiversity movement in which verbal and cognitively able autistic adults advocate for the significance of their experience and its qualities. The previous exclusion of these accounts can now be seen as a form of epistemic injustice (Fricker, 2007), as well as arguably having had the effect of narrowing scientific and clinical notions of what autism might be, with consequences for clinical neuroscientific accounts and research agendas. A new empirical autistic phenomenology now seems both necessary and feasible within a new phase in the historical evolution of the autism concept, re-attending to the phenomenological but doing so against a background of greatly more information about the normal range of development in brain and mind from neuroscience and developmental psychology research. Moving on from early functional notions of autistic withdrawal and defence, we are now able to frame instead the phenomenological impact of developmental brain differences, ranging from the subtle to the severely disruptive. Additional theories to explain the differences between autistic and non-autistic experiences have come from autistic scholarship, particularly around the social transaction and aspects of attention (e.g., Murray et al., 2005; Milton, 2012; Milton et al., 2022). Such conceptualisations, informed by the personal life experiences of autistic writers, are also beginning to have an impact on autism science. Theoretical directions and collaborations between autistic and non-autistic researchers are starting to be evident in contemporary scholarship on social interaction (Morrison et al., 2020), masking (Pearson and Rose, 2021), and participatory community research (Pickard et al., 2022).

Alongside, there has been a recent acceleration in editorial and position statements advocating a return to a phenomenology approach and phenomenology studies in psychiatry and psychology generally (Stanghellini and Broome, 2014; Kyzar and Denfield, 2023; Ritunnano et al., 2023) and in autism, specifically (Zahavi and Parnas, 2003; Nilsson et al., 2019). Phenomena around a set of primary defining experiences foundational to being can be distinguished within more general descriptive accounts with a broader focus that includes many general aspects of a lived life in society, workplace, and family. Particularly relevant to this paper is a phenomenology that foregrounds these primary distinctive aspects of autistic experience in a way that would, for instance, provide specific differentiation between autistic and non-autistic states and be relevant for autistic delineation. Nevertheless, as Boldsen (2021) notes, even such foregrounded essential autistic experience will always be situated; it remains important to include the social-spatial-material fields of the context within the interpretation. Much current phenomenological work published in autism studies contributes important evidence on the lives of autistic people and their needs with a broad focus (e.g., on social policy, education, and care) (DePape and Lindsay, 2016; Howard et al., 2019; MacLeod, 2019; Williams et al., 2019; Pellicano et al., 2020). There is, however, emerging work on what could be considered more primary essential experiences. It is immediately apparent that one of the features of this recent phenomenological work is how it pushes back against recently received notions within the autism behavioural phenotype and associated cognitive research. For instance, a substantive body of work (Shanker, 2004; De Jaegher, 2013, 2021; Fuchs, 2015) critiques the previous explanatory notion of inter-subjectivity in terms of individuals' theory of mind deficits, introducing alternative framing and evidence on embodied experience in cognition and intersubjectivity, and accounts of autistic selfhood and self-experience that echo some of the recent discussion in schizophrenia (Sass and Parnas, 2003). New participatory methodologies for investigating such experienced inter-subjectivity are also described (De Jaegher et al., 2017). These accounts provide important theoretical discussion and illustrate how a phenomenological perspective can open new conceptualisations and scientific areas for study.

Other recent phenomenological writing takes a turn convergent with empirical evidence from contemporary neuroscience, which has moved beyond a search for specific deficits in the “social brain” to understand a more system-wide set of dislocation differences in early development. One summary of contemporary theoretical and empirical work (Barale et al., 2019) highlights aspects of the “Life World” of autistic people, drawing out common but inconsistent differences in the fundamentals of the experience of “temporality, trans modal perception, organisation of predictability and ‘forward thinking', affectivity and reciprocity, intentionality and praxic coordination”—a complex of differences in experience supported by recent developmental science (Jones et al., 2014), including what can be characterised as the “sensorium”. They make the point that a search for unitary core *deficit* within autism is now past the point; it is more that the integration of multiple and varying fundamental aspects of being is different and confers vulnerability within physical as well as inter-subjective contexts (Barale et al., 2019). In this vein, Narzisi and Muccio (2021) highlight work on altered perceptual and sensory sensitivity and reduced “priors” in cognition, and Rizzo and Röck (2021) describe the “Intense World Theory” of autistic experience, emphasising perceptual and sensory differences, and the consequent delights, capacities, and vulnerable difficulties associated with it. More focused recent qualitative studies using empirical methodology have addressed autistic sensory experience (Sibeoni et al., 2022; Taels et al., 2023), anxiety (Magiati et al., 2017), gender dysphoria (Cooper et al., 2023), and the potential roots of some high level autistic sensory abilities (Ockelford, 2015). Despite this, there remains a relative lack of autistic researcher-mediated work or the direct autistic voice in this literature—something that we now represent further below.

**Figure 1:**
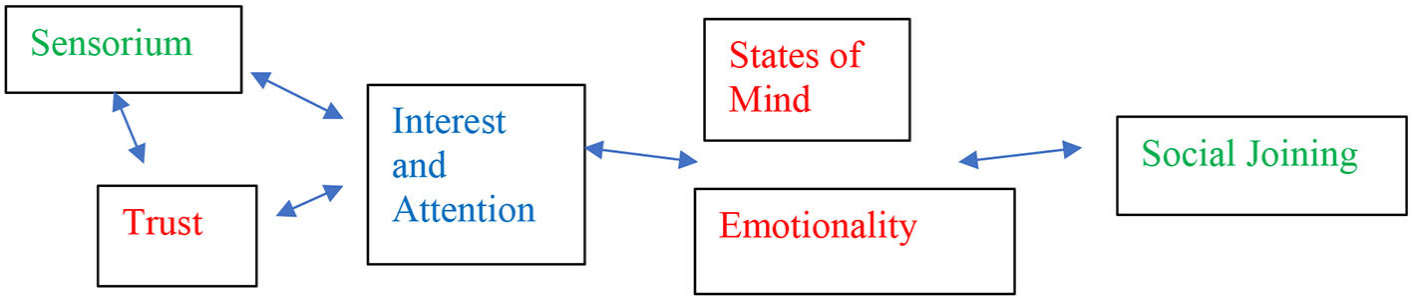
Derived phenomenological themes across autistic and non-autistic experience (reproduced with permission from Murray et al., 2023, available at https://doi.org/10.1159/000526213). Themes showing commonalities (red); differences (green); much overlap (blue).

As a recent step toward a participatory autistic phenomenology, Murray et al. (2023) reported an in-depth comparative peer-mediated phenomenological enquiry across the autism/non-autism divide between three autistic and one non-autistic participants. Many aspects of lived experience were found in common (see Figure 1). For instance, the need for intimacy, trust and acceptance, and the enjoyment of social contact on the right terms; in a way that radically challenges classic notions of a fundamental disconnection from the world or of necessary intrinsic social avoidance and impairment in the current definition, as well as giving insights into common core needs and values across the “human spectrum”. But the areas of difference were also striking, particularly the distinctive experience of the autistic sensorium from early life and the early experience of being overwhelmed by experiences or misunderstood by others. This study echoes Barale et al. in arguing that it is likely that the core sensorium is foundational in the experience and reality of neurodivergence and may provide a key guide as to where neuroscience might look for future insights. A great challenge, clinically and methodologically, will be how to approach equivalent insights for young children and for older people with complex communication needs and to test how much a common experience may or may not underlie the heterogeneity in autistic people and their expression, and we return to this below.

## A contemporary clinical phenomenology

It could be said that the practise of phenomenology concerns particular forms of attentiveness. Classical philosophical phenomenology is concerned with a quality of open attentiveness to one's own response to the world, removing as far as possible prior pre-conceptions to investigate the structure of consciousness and perception. Clinical phenomenology, on the other hand, pertains to the quality of attentiveness to the experience of another in an effort to understand their mind; as this evolved, the idea of clinical phenomenology progressed to describing another's state of mind in illness (Zahavi, 2018). Binswanger's emphasis on the relational, described above, anticipated a kind of attentive interpersonal listening that has become central to psychotherapy and much clinical mental health practise (Frie, 2000). In ideal terms, clinical description reports an encounter between the subject and an experienced and attentive other in a context of confidentially and intimacy. Binswanger spoke of a kind of agape (ethical love) aspired to in such clinical encounters (Hoffmann and Knorr, 2018), exemplifying a general human effort toward imaginative empathy across divides of otherness. Extended case descriptions, such as in Binswanger's of Lola Voss (Needleman, 1968), give vivid evidence of the autism concept of the time, just as have others more recently (Sacks, 1995). Nevertheless, there are inevitable partialities of the clinical context, such as ethical issues in relaying specific experiences while ensuring anonymity, selecting particular clients, or in a particular context in their lives, which may be different to a non-clinical context or autistic self-report. The clinician's accounts are also situated in the context of providing care, which may influence what they attend to and report. They also need to be understood explicitly in the context of the conceptual thinking of the time since, despite all efforts toward phenomenological reduction in setting aside prior concepts, the core organisation of a clinician's perception will still be informed by current ideas. To illustrate this point, it is enough to look back on clinicians' accounts from previous generations. The clinical accounts that follow are subject to all these considerations. They are vignettes, fully anonymised with key identifying details changed, and within which the quotes are illustrative close transliterations rather than verbatim recordings. They are offered to show what a contemporary clinical phenomenology can be like from the positionality of a clinician (JG) who is himself non-autistic and working in a period of neurodevelopmental conceptualisation of autism. This section can then be balanced against the following one of autistic auto-ethnography.

### Inter-subjective dislocation and social difficulties

Two cases illustrate the experience of the *inter-subjective dislocation and social difficulties* spoken of by Barale et al. above. Brian is an autistic young man of 17 whom I met in the context of acute crises of anxiety and suicidality. Brian's main preoccupation is his experienced difficulty in negotiating the regular social world—in peer contact, social graces, and the ability to engage a girl he is attracted to. The phenomenology of his social difficulty is most clearly expressed in a distinction between his perplexity and anxiety at what he calls the “analogue reality” of every day, compared to his mastery of the “digital reality” of online environments. “*When I'm with another person, I just can't tell what is going to happen next, what they mean with this look in their eyes or what they say; I always feel I'm going to make a mistake. When I'm online, I can understand the rules there and know what they're going to do*”. He describes a core difficulty then around the inference of inter-subjective intent (the inferences beyond observed behaviour which make human interactions predictable), in contrast to his ease with algorithm rule-based interactions in the computer game. To be, in Temple Grandin's words, a person who has “to learn by trial and error what certain gestures and facial expressions mean” (Grandin, 1995). Brian is very popular in his peer group; they love him for his attractiveness and the self-deprecating humour he has developed to get by, but he is agonised by the sense he will never be able to have a relationship or family. He became passionately attracted to a girl in his class and then devastated by being unable to comprehend her non-response. In such moments of deep perplexity, he often decompensates, melts down, explodes with violence or worry with impulsive and serious suicidality, and needs to be rescued repeatedly from dangerous situations. As a participant commented in Murray et al. (2023), “*Not being able to bring one's gift to others does deep harm to people's lives*”; echoing Binswanger's formulation of an effective and productive “being-in-the-world” as a necessity condition for mental health. In contrast to what is often implied by the older phenomenological literature as a primary withdrawal from reality into fantasy, the phenomenology here suggests rather that there is a primarily felt dislocation between self and other, with the internal world elaboration secondary. Adapting the external environment to be more in keeping with neurodiverse abilities and perceptions may mitigate this gap, but the depth of difficulties and normative aspirations for relationships remains a vulnerability (Barale et al., 2019). Brian is able to establish a bond with me within the adapted environment of a therapeutic encounter, and this helps his emotional self-regulation and suicidality. The task is to find a way to help him locate himself in the world (and the world to him). Practical strategies and specific supports can be rehearsed and go so far; there is a certain kind of existential adaptation that is needed to differentiate personally felt and socially normed expectations. With many autistic young people emerging into adulthood, the fundamental task is this accommodation to identity, adaptation, and effectiveness, given developmental differences. Brian does well and grows in confidence to the extent that he is able to take up a long-desired place at university. Great efforts go into constructing an adapted support network for him there, but sadly, it breaks down in the first semester; he is unable to manage life away from home in halls of residence and has to break off his studies.

William was an autistic teenager of around 16 years with normal range cognition and language/communication ability who presented in the late 1980s with an acute mental health crisis related to anxiety, depression, and suicidal intent. I began work with him at that time using an adapted form of insight-orientated psychotherapy that I was using with other non-autistic teens. His father owned a furniture workshop, and it was his father's but also William's fervent wish as the eldest child to follow him into the family business. William became able to talk about an extraordinarily intense fantasy life, filled with the construction of a detailed imaginary space of an idealised workshop that he owned. He imbued it with socio-political ideals as a perfect world where workers and managers existed in harmony and that he would personally own and develop as he took over from his father. The workshop was laid out in vivid detail in his mind in spatial organisation, structure, and working relationships. Huge amounts of his time were spent organising details down to written worksheets, protocols, contracts, and vignettes about the working life and the development of the product line.

As William got older, his father, concerned, gave him a role in the workshop as a store handler and warehouse boy. For William, anything related to social contact in the workshop caused him a huge amount of difficulty, as well as humiliation, in terms of his idealised future. The gap between his social life in the workplace and the leadership role in his imagined workshop was exquisitely painful.

I began with techniques of insight-orientated therapy, aiming to use reflexivity and interpretation to link affect and thought through insight and work through realities about his position in the family, his likely capacities in future, and his ambivalent relationships with his parents. But it became clear that a core experiential difficulty for him was *just this linking*. It provoked a frantic perplexity and anxiety in the realisation of knowing that he did not know something but not knowing why he did not know it. Exploration around this, within what was manageable for him, just served to show how central this was in his autism. It revealed how difficult it was to approach and how much it showed characteristics of neurological difference rather than psychological reaction or defence (a key phenomenological distinction for clinical work). On the other hand, his skillfully elaborated internal world defied accepted views of the time on a developmental lack of capacity for internal fantasy in autism, instead being what can now be understood as an intense monotropism. In contrast to early theorising that such fantasy represented a primary pathology, this internal world elaboration appeared more to serve the function of rehearsal, meaning-making and problem-solving—his alternative to conventional insight.

I came over time to work more with the grain of this imaginative fantasy and his autistic thinking rather than with insight as the mechanism for change and problem-solving. Working together over several years, covering his late adolescence and painful adult emergence, reduced his suicidality and led to a partial accommodation on behalf of both him and his family. As his father became more empathic and realistic (he had not accepted the autism diagnosis), the whole family structure became more reality orientated, and this began to help his distress. His personal experience was thus situated within a pattern of family and social expectations that needed understanding in order to help his development and work within his internal imaginative world; increased empathy from his family was his means to this. Such phenomenology around adult emergence is shared by many autistic teenagers—difficulty in working out implicit social rules and the perplexity that this involves, particularly in early adolescence when the child becomes more aware of complex social interaction; a sense that everyone else is in on the joke, and a phenomenological experience of opacity, of being unable to grasp something that they feel is implicitly apparent to others, and a painful sense of being out of step.

### Sensory sensitivity

Sally is a 13-year-old autistic girl who was brought by her parents with longstanding concerns about anxiety, which had led to her being often homebound, avoiding outside situations, and not being able to go to school, although she is a talented team athlete. Her parents were sure that coming to the clinic would be disastrous and that she would not leave the car, but she did agree to come in. We spent the day with her; a multidisciplinary professional team, including psychiatrists, psychologists, nurses, speech therapists, and occupational therapists, focused on understanding her needs. On technical testing, she had an average range cognitive ability but, similar to many autistic children, a spiky profile of abilities within which we were able to understand in detail high levels of skill in quick and efficient visual processing but a much slower ability to sequence and process auditory and verbal information in her short-term memory. Her capacity to understand emotion and “theory of mind” was average for her age. When I talked with her, Sally is completely preoccupied with managing her sensory life every day. She feels frequently overwhelmed with disorientation in balance, coordination, and a sense of herself in space, in working out sounds and language. She loves movement as a way of dealing with aspects of her life that are more difficult, but without clear visual input (for instance, when on an escalator), she feels vertiginous and panicky. She hums and sings to drown out sounds when she finds them uncomfortable and panics about following verbal instructions.

She can talk about anxiety feelings throughout the day, particularly with change or uncertainty, and two fundamental experiences related to this. Firstly, her constant sense that—particularly in complex situations, both social and non-social—“*I just can't work things out quickly enough, and then I can't manage*.” It is the sense that the world is going too fast in the moment for her to process things in time, related to her slower processing and working memory; “*I can't slow down or stop to work things out—everything's going on anyway and won't slow down for me—and then it all goes wrong*.” Secondly, in those moments, she then gets a cascade of anxious thoughts about remembered and anticipated catastrophic outcomes, panic rises in her body, and she must avoid and withdraw. On the other hand, there are situations, particularly in athletic classes, in an environment with people she knows and a physical routine that she understands, where she can get into a familiar movement flow and feels comfortable, confident and even ecstatic; a point where “*I can keep up with myself* ” in movement in time and space, and everything flows together (“flow states” of this kind are also discussed in Murray et al., 2023, p. 4).

We understand that over time and experience, these fundamental experiences have got overlaid with so many anxious worries and resistant avoidant management behaviours that their source is difficult to discern. Such disruption of the sensorium is commonly described in many autistic experiences, in the difficulty to integrate sensory experience in space and time, resulting in discomfort, disorientation, then a breakdown of sense of being and huge anxiety. And yet, in an environment where the balance is found, there can be the ecstasy of a flow state and the relief of integration. When the experienced pace and demands can be slowed, and trust in the environment is found, Sally is able to relax. Within a therapeutic relationship, she begins to be able to understand her sensory experiences. It becomes possible for her to construct better ways of managing her own functioning, gradually increasing her confidence within the experience of being autistic.

For another 13-year-old girl, Tina, the pattern of sensory differences is subtly different; here, there is a slowness in registration of sensation, needing more time to process, meaning that she has difficulty reacting to rapidly presented or low-intensity stimuli, and she often needs to seek sensory input to keep focused. At some times, she finds auditory inputs hard and uncomfortable; at others, she instead seeks auditory input and likes having music around. During the interview, she talks about her sensory experiences, particularly at school, where she finds it very difficult coming down corridors with other students, overwhelmed with sounds and bustle that she cannot cope with. She has learned at these times to “space out” instead of getting upset—“getting upset would help me let other people know what was wrong, but spacing out ends up bottling it all up. When I space out, my attention goes, and I don't feel myself. Usually, I feel myself strongly, but when I space out, the sense of who I am goes.” “I really like painting and drawing, and if I can just slow down and not be rushed, then I can make sense of things.” For this girl, when her capacity to process and be in the world in space and time falls apart, she either gets very anxious or dissociates (spaces out), which works in the short term but cuts her off from everyone. She has anticipatory anxiety, which leads her to avoid school. Working with our therapist on understanding and managing these situations goes on for several months and is helpful. She can manage her sensory world more confidently now, and although things were up and down, she feels better about dealing with everyday activities most of the time. She is now able to go out with her family to restaurants and make a beginning at college since her obsessive checking and concerns about cleanliness and food have disappeared.

These case studies of autistic girls and sensory features are also suggestive of how sensory features are foregrounded as a core characteristic. These findings are consistent with recent phenomenological studies of sensory experience (Sibeoni et al., 2022, and particularly Taels et al., 2023) and work on autism and gender (Osório et al., 2021).

### Children with complex communication needs

In a specialist school, I spend time with David, a 10-year-old boy with a significant learning disability and high-level needs—preverbal and within the very early stages of the individual curriculum. He is a boy showing high frequent levels of intense distress behaviours, self-harm, lashing out, screaming, and crying that do not necessarily have any clear environmental trigger. The challenge is to work back from these behaviours to understand something of his underlying internal experience, whether sensory, affective, or cognitive. By interacting with him I want to find out what gives him pleasure, what gives him distress, and in what sense he may be able to connect and use me as a comfort. I spend repeated time just lying beside David or in rough-and-tumble with him, watching for and mirroring his behaviour and reactions, waiting to find moments of reciprocity when my response to him leads to his response to me, the smallest signals in other words of reciprocal connection. From this, I try to infer the nature and quality of his experience and capacity, and also give him a sense of reciprocal relatedness. I can describe fleeting moments of reciprocity and relaxation, synchronised to and fro with transient pleasure and recognition; before his behaviour again settles away into something that feels out of reach.

## Contemporary autistic accounts

A core feature in autistic life writing is the concept of the *sensorium* (Green, 2022; Murray et al., 2023), which interacts and intersects with several other thematic characteristics, particularly *attention differences* (monotropism, Murray et al., 2005; Murray, 2019), *sociality* (double empathy theory, Milton, 2012; Milton et al., 2022) and *ontology*, an existential theme that can be positive or negative. Its positive form is associated with a vivid sense of reality and presence in time and space, while the negative aspect involves intense overwhelm and undermining a person's sense of being in the world, the reality of their sense of self, and their relations with the environment and others.

These themes can be seen in a diversity of autobiographical texts by autistic writers whose lived experiences encompass gender, race, and cognitive differences. For the purposes of this study, examples are drawn from four authors representing different perspectives (in terms of gender, ethnicity, and disability) and whose writing provides detailed accounts of childhood experiences: *Born on a Blue Day* (Tammet, 2006), *The Reason I Jump* (Higashida, 2013)*, The Secret Life of a Black Aspie* (Prahlad, 2017), and *The Electricity of Every Living Thing* (May, 2018).^2^ The collation is done by an arts-based scholar (NS) who identifies as autistic and neurodivergent.

### Sensorium

The intensity of sensory experience and awareness is a prevalent and often defining theme of autistic life writing, indicating its significance for an autistic consciousness of being in the world. In Tammet's memoir, *Born on a Blue Day*, synaesthesia is part of the sensory textures. Numbers are described as a “first language, one I often feel and think in” (Tammet, 2006). For Tammet, reported to be “absorbed in my own world”, other children are remembered as “the background to [his] visual and tactile experiences”. Many aspects of Tammet's description of early years are characteristic of archetypal features of early autism as he recollects his fascination with shape, colour, objects, and patterns in memories of coloured beads, the “shape and motion” of blowing bubbles, his “obsession with hourglasses” and their “trickling flow of sand” (Narzisi and Muccio, 2021), and “taking a coin and spinning it on the floor and watching it go round and round” (Murray et al., 2023). This, however, is a memoir of a savant diagnosed with Asperger syndrome as an adult, whose abilities and experiences are radically different to most autistic people. As Tammet acknowledges, “I didn't rock my body continuously; I could talk and showed at least some ability to interact with the environment around me” (Tammet, 2006).

Intense sensory engagement is similarly fundamental to Higashida's articulation of autistic experience and his sense of being in the world differently. Like Tammet, he enjoys spinning objects. “Everyday scenery doesn't rotate, so things that do spin simply fascinate us. Just watching spinning things fills us with a sort of everlasting bliss-for the time we sit watching them, they rotate with perfect regularity” (Higashida, 2013). This is sensory stimming, a key facet of repetitive autistic behaviour, also associated with sensory regulation and flow. “By performing whatever action it is, we feel a bit soothed and calmed down” (Higashida, 2013, p. 139). Descriptions of the pleasure and need for stimming as a means of sensory integration reverberate throughout autistic writing, creating temporal and spatial order in environments that threaten chaos.

For Prahlad, like Tammet, autistic sensory experience intersects with synaesthesia: “People talk to me, and they assume I'm hearing and understanding their words. But usually, I'm listening to their colours. I'm seeing them. I'm feeling their temperatures. I'm smelling their scents. And whatever I see or smell or touch, I taste” (Prahlad, 2017). One of the distinctive features of Prahlad's autistic experience is difficulty with memory due to not thinking in pictures: “I think more in feelings and senses. In colours and sounds […] Not remembering things might have something to do with not seeing images in my mind. If you said, ‘Imagine a cat,' I would imagine the way cats make me feel. But I wouldn't get a picture of a cat” (Prahlad, 2017).

Imagination is a feature of the autism phenotype that needs more research and more nuanced understanding, as many autistic people also experience *aphantasia*. Nevertheless, Prahlad's earliest memories are sensory and vividly detailed (detailed perception being a creative strength)—“the blossoms of a redbud outside the trailer window. I was stunned by their bright pink, pulpy stillness. I thought I was one of them. I thought my body hung somehow in sea blue, a cluster of soft petals, suspended and still, floating in space” (Prahlad, 2017). This sense of oneness with nature is also a common feature of autistic life writing.

Katherine May's *The Electricity of Every Living Thing* narrates her experience of late diagnosis and shifting understanding of her personal history alongside a year-long series of walks as a psychological and physical journey. The electricity motif is inextricably related to the sensorium—“My world is made up of tiny electric shots. Every living thing carries its own current, and this finds its earth through me. Every unexpected touch, every glance, has a charge. I am a lightning rod, …., eternally braced for the metal-on-metal jolt of contact” (64). Electricity is both a positive and negative force, “people carry electricity for me; they have a current that surges around my body until I'm exhausted…unpredictable demands make the air thick, like humanity has…not a scent, but a texture. It makes me feel like I can't breathe” (9). It is also a maternal force. Her son is “the only person in my life whose electricity exactly matches my own, whose touch is as native to my skin as air or water” (245). Electricity for May is often triggered by sensory encounters, but touch is particularly acute and often negative.

In *The Reason I Jump*, Higashida explains touch sensitivity that is consistent with its representation in autistic texts and certainly provides a strong connection to *Electricity* (despite the radical differences between these authors in terms of gender, age, and autistic experience)—“being touched by someone else means that the toucher is exercising control over a body, which even its owner can't properly control. It's as if we lose who we are” (55-6). This, again, is an indication of how the sensorium intersects with ontology.

### Attention differences (monotropism)

The sensorium is inextricably linked to attention differences in autism, and this is particularly evident in autistic writing and scholarship. Monotropism is a term used to conceptualise thinking as being shaped by focused interests, flow states, and attention to detail (Murray et al., 2005; Murray, 2019). This theorisation of the mind as an *attention tunnel* (Murray, 2019) is modelled on the capacity for autistic thinkers to become hyper-focussed on a particular topic or preference and for this to direct their attention almost exclusively. Sensory differences are also linked to monotropism, as Fergus Murray articulates: “If we can't tune an input out, it is often experienced as horribly intrusive… neural pathways that receive a lot of stimulation grow stronger, so perhaps autistic people are prone to long- term hyper-sensitivity in senses receiving intense attention, and under-sensitivity in channels we regularly tune out” (Murray, 2019, p. 46).

This is richly evoked in autistic writing, as in the immersive pleasure of Prahlad's redbud memory. In a particularly vivid memory of his grandmother's pantry, there is an evocative entanglement of sensory experience and detailed perception:

“*The jars of jams sang and talked to me… One* of my favourite things to do was to watch flecks of dust floating in streaks of light or sunshine. … *The door was made of vertical rows of boards, painted light yellow on the outside, like washed-out mustard, like saffron or daffodils mixed with cream*” (emphasis added) (26-7).

Detailed perception is similarly celebrated in *The Reason I Jump*:

“*When you see an object, it seems that you see it as an entire thing first, and only afterward do its details follow on. But for people with autism, the details jump straight out at us first, and then, only gradually, detail by detail, does the whole image sort of float up into focus*” (91-2).

Detailed attention can also be experienced negatively, particularly in relation to sound. Prahlad refers to his experience of restaurants: “I can't stop hearing all of the conversation around me, or the patterns of silverware striking glasses and plates. […] And so, I hear only bits and pieces of what the people I'm sitting with are saying” (Prahlad, 2017).

Attention to detail is linked to intense interests. In *Electricity*, May refers explicitly to the need for a deeper understanding of this autistic trait: “*Detail has nuance. Detail has application. Not all detail is iterative, blunt, and competitive*. I don't deny that my brain holds detail. I don't deny that it sucks in more detail than other people's brains, making it difficult to navigate simple situations due to an excess of input” (emphasis added) (p. 248). May describes the detail of her maternal understanding of her new-born son's needs and cues: “he would click his little tongue kkkk—in the seconds before he would start to wail his hunger […].” In this “call and response” attention to detail, “the wonderful and terrible pull of motherhood”, she emphasises that she is “not claiming any special powers” [as] those details “make me more like an ordinary woman than I've ever felt in my life before” (p. 250). However, the converse is worth considering as it may well be the case that for the ordinary non-autistic woman, the intensely detailed focus of the mother and baby relationship (positive or negative) is, in fact, a coming closer to the intensity of autistic experience in terms of detailed attention (monotropism) and a focus on survival.

### Empathy and sociality

It is very evident in autistic autobiographical texts, as in the human spectrum paper (Murray et al., 2023), that characteristics long thought intrinsic to autism, such as social misperception and reduced empathy, may be alternatively understood as state-dependent outcomes contingent on specific contexts and interactions. May, in *Electricity*, enjoys loving relationships with her husband and son and has a network of friends who accompany her on different stages of the coastal walk. She has a very high level of empathic understanding, is attuned to the feelings of others, and connects this to detailed perception: “Detail doesn't only lie in systematic knowledge of football cars or aeroplane serial numbers. There are other kinds of detail too […] there is the detail—quite mysterious to me—of noticing someone else's mood and knowing what will draw them toward their equilibrium” (p. 248). Higashida is reported to reiterate repeatedly that “he values the company of other people very much. But because communication is so fraught with problems, a person with autism tends to end up alone in a corner, where people then see him or her and think, ‘Aha, a classic sign of autism, that”' (Higashida, 2013).

Tammet acknowledges the respite in solitude, the sense of outsider status and the difficulties of engaging with his peers:

“*Slowly, I think the feeling was creeping over me that I was different from the other children, but for some reason, it didn't bother me. I didn't yet feel any desire for friends; I was happy enough playing by myself. When the time came to play social games, such as musical chairs, I refused to join in. I was frightened by the thought of the other children touching me as they shoved one another*”.

May similarly describes her sense of being an outsider and “poor at taking the social temperature” (May, 2018), particularly in childhood. In a passage that strikingly resonates with Tammet, she writes:

“*I had few friends as a child, and although I wouldn't say I liked it that way, I can't say it bothered me much either. I liked my own company. The painful part was being conscious of my own difference. I was another species entirely from the little girls in my class, with my big, awkward body and complete inability to relate to anything they said or did*” (p. 47).

Autism being experienced as speaking a “different language” with a need for translation is also a recurrent motif that has been theorised by Damian Milton in terms of a double empathy problem (Milton, 2012), with a mutual failure to understand both ways of communication between autistic and non-autistic people, as different neurotypes.^3^ For May: “I did everything I could to speak the same language as them, but I could see that it landed differently. I felt like a wild-eyed beast who speaks beautiful words, only to find them received as grunts and snarls. There was a translation error somewhere down the line” (May, 2018).

Autistic life writing offers evidence of how “a different embodied way of being” contributes to “effects on social interactions and understanding” (Milton et al., 2022) so that autistic sociality and community are prevalent and positive themes in this corpus of writing. Given the emphasis on intersubjectivity and early relations with caregivers in discussions of autism, it is noteworthy that in autistic writing, we frequently find examples of attention, attunement, and joy. Prahlad expresses one of the earliest memories of his mother's face as: “A maple-brown expanse of garden glowing, with lips that moved on her whispers and breath like a butterfly's wings when it sits on a blossom” (Prahlad, 2017). The engagement may be different, as Higashida explains in relation to eye contact: “What we're actually looking at is the other person's voice. Voices may not be visible things, but we're trying to listen to the other person with all of our sense organs” (Higashida, 2013). For many writers, neurodivergent parents are alluded to. For instance, Tammet's mother “had always considered herself an outsider as a child”, and he describes the pleasure of sitting on his parents' laps (who were dedicated readers) and enjoying the sensory aspects and shared pleasures of books as “the room would fill with silence” (Tammet, 2006).

The complexities and dynamics of empathic engagement and disengagement are fundamental to the tensions inherent in autistic texts. In May's memoir, this is connected to detailed attention:

“*I over read other people's feelings to the point that they choke me…As a child, other people's emotions seemed to come out of nowhere. There would suddenly be crying or hysterical laughter, and I would flounder, wondering how on earth we got here*” (p. 208).

A corollary of the double empathy problem is the extreme state of physical and psychic distress so frequently induced by sustained or intensive exposure to social space. In *Electricity*, these episodes punctuate the text like missiles, reminding the reader of the painful and felt reality of being neurodivergent in a non-autistic world.

“*I realise the whiteness has started […] a multiroom cloud of searing blankness. It feels like someone has pushed a knitting needle through my skull […] maybe their whole hand is in there palpating the matter it finds inside, squeezing the sense out of whole regions of my mind*” (92).

### Ontology

Prahlad refers to “always feeling disconnected” (4). This goes to the heart of the ontology theme, the existential need to feel a connection (to the environment, nature, people, and reality) and an associated experience of alienation. This ontology theme is similarly pervasive, often entangled with the sensorium and is also frequently associated with sociality, particularly when overwhelm is triggered by a social environment. A questioning of the reality of existence, feelings of disembodiment and the pull toward the natural environment (often seeking immersion) are shared features. This can take the form of self-harm, and it is striking to see head banging referenced so frequently—“at age two, I began to walk up to a particular wall in the living room and band my head against it. I would rock my body backwards and forwards, striking my forehead hard, repeatedly, and rhythmically against the wall” (Blue Day, 23). Tammet's reference (vestibular stimulation) connects to Higashida's jumping, which, in turn, links to the pull toward nature that can be observed across autistic texts: “When I'm jumping, it's as if my feelings are going upwards to the sky. Really, my urge to be swallowed by the sky is enough to make my heart quiver” (Tammet, 2006). The power of auditory perception, positively and negatively, is a core element of the ontology theme. As Higashida explains, “There are certain noises you don't notice, but that really gets to us… It's not quite that the noises grate on our nerves. Its more to do with a fear that if we keep listening, we'll lose all sense of where we are” (Higashida, 2013).

### Reflection

Whilst there is considerable value in this rapidly developing corpus of autistic life writing (contributing to what is sometimes referred to as “autie-ethnography”; Yergeau, 2013), there are limitations that need to be highlighted. There is a long tradition of literary criticism foregrounding narrative methods in autobiography and memoir, questioning the truth status of the genre and conceptualising critical autobiography (Di Summa-Knoop, 2017). Like all life writing, autiebiography (Van Goidsenhoven, 2017) offers subjective perspectives that are necessarily selective in several ways. We need to be aware of issues of voice and representation in terms of who can write these accounts. The vast majority represent the perspectives and experiences of educated, articulate, and white autistic people. In *The Reason I Jump*, the form and style (questions and responses) are different to most autistic life writing. This dialectic account, foregrounding editorial collaboration, can be considered in the context of current practises of co-production (and in relation to more established methods of assisted communication). It is also worth noting the prevalence of autobiographies by women writers, which does not reflect the history of gender bias in diagnosis. Other factors concern the contexts in which books are produced and the publishing process. There is the influence of the selection criteria for publishers, the potential distortion through editorial intervention, as well as the prism of ghost-writers. Moreover, cultural preferences are also at play in terms of the vogue for overcoming illness and success narratives. A further limitation is not having a readily identifiable comparative element. Although autistic life writing and clinical phenomenological accounts allow rich insights into subjective experience, they are part of a complex picture framed by different lenses. They provide depth and vividness but are limited in several ways in terms of breadth.

## Toward a systematic autistic phenomenology

The great majority of formal autistic phenomenology literature to date has been clinical phenomenology, originating in professionals' experience within clinical care. Direct autistic voice has been notably absent, represented in this paper by the preceding section. Going forward, we propose the need for a more systematic, empirical, and representative approach to autistic phenomenology, echoing calls from others in the field (Nilsson et al., 2019). This is based on the view that what has been the essentially clinical case-report idiographic methods previously used need to be supplemented with large-scale representative methods, without sacrificing the deep and intimate subjective qualities of traditional phenomenology. The methodological challenges of doing this will be substantial, but the goal is ambitious—a reframed autism concept which includes the subjective and phenomenological, and one that may re-vitalise diagnostic language and provide new impetus to scientific enquiry through more focus around autistic experience.

Several principled stages in this approach can be envisaged:

**Co-construction:** Such a project must be co-constructed collaboratively between often non-autistic researchers and autistic people themselves because, theoretically and practically, this collaboration will be necessary and because the absence of the autistic voice in a true autistic phenomenology constitutes a form of epistemic injustice, which needs to be corrected. There is now an evolving methodology to draw on Pellicano and den Houting (2022) and Shaughnessy (2022) in which autistic and non-autistic researchers can find ways to collaborate on equal terms in ways that address concerns about inequalities of power and representation in such activity, building on good practise in participatory research.

**Thematic analysis of existing accounts:** This would begin with a collation (as we have begun in this study) of descriptive observation, clinical case studies, and phenomenology studies, along with autie-ethnographic first-person accounts of life experience within the autistic literature. Such a collation and review could then allow a descriptive analysis of common elements and themes across such diverse literature. Such analysis could serve to initially orientate the work and highlight foci and sampling strategy for the next stage of a more purposive, in-depth study of autistic consciousness and life experience.

**Idiographic study:** In-depth idiographic exploration for key themes, necessarily on a relatively small cohort but with purposive sampling. Various applied phenomenological methods could be utilised. The most empirically inductive might be semantic analysis techniques, such as in Petitmengin et al. (2018), taking narrative data from transcripts and encoding them into empirical clusters using inductive techniques, rather in the same way as natural language processing in artificial intelligence machine learning. Other approaches, whether based on transcript or interview, could be based more on a degree of inter-subjective meaning-making by the interviewer and/or interviewee. These might include the “descriptive phenomenological psychological method” of Giorgi (2012). Alternatively, it could include an “interpretive phenomenological analysis” (IPA; Smith et al., 2009) where each person's account receives systematic interpretative understanding in its own terms, using what Smith describes as a double hermeneutic where “the interviewer is trying to make sense of the participant trying to make sense of their experience”. Thereafter, convergences and divergences between individuals are explored.

Whatever the methodological technique is used, there will be common (and challenging) issues to consider. Firstly, building on the principle of co-construction, there will need to be an approach to autistic participants that is carefully consensual and sensitive to the potential power and experiential differentials in such engagement. The involvement of the autistic community and autistic researchers in this work will be central to its acceptability and value. Secondly, a sampling strategy for these intrinsically small sample studies will need careful consideration, including key intersectionalities of, for instance, age, gender, and ability, and building on the prior thematic review. This will be necessary to begin approaching representativeness across autistic heterogeneity. Thirdly, an equivalent sampling from non-autistic experience will be arguably crucial. Only with this will one be able to establish the specificity and discrimination for autism itself of the accounts made. On a small scale, the work of Murray et al. (2023), illustrates the insights that can arise from doing this.

**Survey breadth:** To achieve the necessary breadth required to inform a representative autistic phenomenology, the next step would be to iteratively to combine the in-depth idiographic data above with a broader approach to data collection offered by online digital technology platforms, such as those employing a citizen science approach. Citizen science, as a highly diverse practise, is already being used to enable people to contribute to research, both through crowdsourcing the collection of data as well as the processing and analyses of larger data sets (Haklay et al., 2021). While online citizen science has not yet been applied to autism research at scale, it would be highly applicable for an “in-breadth” phenomenology of this kind. Research has already found that the structured format of existing social media sites can give autistic users the chance to contribute without having to worry about how they are being perceived (van Schalkwyk et al., 2017), enabling the large-scale collection of autistic people's experiences. Themes and sub-themes capturing significant topics identified from analysis of the in-depth accounts would be used to generate specific prompts inviting autistic and relevant comparison groups to contribute their own related experiences at large scale across intersectionalties such as age, gender, and ethnicity. Data gathered via an online platform, in turn, will provide a test of the validity of the original, in-depth accounts. Such an approach could be used in conjunction with automated methods of analysis using machine learning/AI methods, such as Natural Language Processing (NLP) models, as well as other forms of qualitative analysis, such as content and framework or template analysis. In this way, a more representative account will be built, reflecting the diversity as well as the specificity of autistic subjective experiences and their similarities and differences to the experiences of relevant comparison groups. The big data sets potentially generated would allow the power for a stratified analytic strategy to investigate formally the specificity and representativeness of phenomenology across many sub-groupings of autistic experience.

**Representation across ability:** A key methodological challenge will be that of representativeness across ability, particularly within intellectual disability and participants with complex communication needs. Accounts within clinical phenomenology and autistic auto-ethnography almost wholly represent the experience of verbally and intellectually able autistic people and constitute, in Jaarsma and Welin's terms, a “narrow neurodiversity” concept (Jaarsma and Welin, 2012) in contrast to a “broad neurodiversity”. While this is an essential beginning point to establish the parameters of autistic experience, we then need to have a method of testing out such constructs within the range of intellectual and language ability associated with autism. A key question here is whether an autism concept or experience is relatively continuous across these variables (the epidemiological, behavioural phenotype evidence suggests that it is, hence, the disaggregation of autism from IQ in current clinical nosology), and systematic phenomenology should have at least a role in clarifying this issue. Methods to be developed and validated for this could include new neuroscience methods (e.g., eye-tracking to signal intent and communication) for augmented or alternative communication, the use of ethnographic and participant observation methods such as PRISMA (De Jaegher et al., 2017), and related creative practises. They will allow us to test out or explore, beyond verbal communication, core elements of autistic people's experience. Such work will be particularly important if the outcome of the work is to influence clinical nosological definitions and to guide clinical neuroscience investigation since such differences and disabilities are key within much healthcare intervention.

**A developmental perspective:** Another central issue in interpreting subjective statements about autistic phenomenology will be its developmental nature. Does what one is tapping into constitute a primary aspect of autistic difference or the developmental outcome of a cascade of self-environment interactions over time? This is going to be a complex issue to work out, but one that epidemiology in other areas has methodologically addressed. One beginning will be the careful reconstruction of developmental experience in a sequenced fashion, for instance, using large datasets classified by age, and this will begin to help the discrimination of early experiences from later secondary ones. Exemplifying this process is evidence from reported phenomenology in early development (Murray et al., 2023), supported by results from early intervention science (Green, 2022). This evidence is convervent with more theoretical literature in suggesting that at least some of the social differences and impacts central to the currently described autistic behavioural phenotype are, in fact, secondarily emergent rather than primary. In other words, these differences may arise from transactions between neurodiverse individuals and their environment in the early years.

## Discussion

This study contains and juxtaposes several representations and expressions of autistic experience manifested over time and across across perspectives: (1) in the early years of phenomenological psychiatry, usually within the context of schizophrenia; (2) as conceptualised within early developmental science; (3) as theorised in terms of recent clinical and scientific reviews; (4) as experienced in a current clinical context; and (5) as described by the voices of autistic people writing about themselves. These juxtapositions set up a series of resonating comparisons across historical time and between perspectives. The contemporary autistic voice, for instance, resonates in some striking ways with the early clinical phenomenology in descriptions of highly intense, elaborated and absorbing *internal worlds*. The quality of these descriptions, however, varies. They are framed pathologically in the early clinical accounts, linked, for instance, to analytic drive theory or a loss of being. They are framed more creatively and positively in contemporary accounts of autistic sensitivity, intuition, and hyper-awareness, although with concomitant vulnerability to being overwhelmed by environments and experiences. The contrasting idea from some early developmental science literature that the slower language and symbolic play development emphasised at that time^4^ was linked to an absence of internal-world fantasy is balanced by suggestions from some contemporary autistic accounts of very rich non-verbal sensory internal environments (such as auditory or visual); difficult to access but part of “spiky profiles” and enhanced sensory abilities in many autistic children (Ockelford, 2015). The re-introduction of interest in the phenomenology of these internal world experiences and potential abilities has substantial potential implications for developmental science concepts (see Evans, 2013).

Similarly common across history and theoretical positions, there is a described quality of *dislocation* between the autistic self and what is felt as a non-autistic reality. This is described from both clinical observation and subjective accounts of a sense of living in a parallel world—understandable within itself but subtly disconnected from a less understandable neuro-typically shared experience (Humphrey and Lewis, 2008). What varies radically across historical time is the causal attributions made to this disconnect; early on, it is understood as driven by absorption in primary pathological fantasy and with a sense of shared sociability needing to be an achieved skill; later, as driven by a “poorness of fit” with or lack of adaptation *from* as well as *to* the neurotypical world. The profusion of acute sensitivity and openness manifest in many of the autistic-voice accounts in this study is a corrective to the intrinsic pathologising of earlier accounts. Yet, there is also a recognition of vulnerabilities and the perplexity associated with encountering a neurotypical assumptive world, which Murray et al. frame as a difficulty with “social joining” (Murray et al., 2023). This (mutual) perplexity resonates with some of the qualitative literature and comes across in contemporary clinical phenomenology.

One can perhaps generalise, in other words to an autistic experience characterised by a sense of difference or dislocation from the shared experience and communication between others, plus experiences of internal world difference and sensitivities, that under some circumstances, can confer strength and benefit and in others can contain a vulnerability to overwhelm and meltdown. This resonates with more general notions from the developmental science literature, for instance, of “differential susceptibility” and the idea of a continuum of underlying difference coupled with emergent difficulty, as articulated by Bervoets and Hens (2020), Bervoets (2022), and Green (2022). For instance, Bervoets (2022) gives an account of “neuro gradualism” from which the (probabilistic rather than determined) emergence of intersubjective dislocation opens up both potential difficulties but also a space for moral imagination within and between both self and other. Naming autism here is a means toward human understanding rather than stigma and echoes back to the empathic interpersonal stance of Binswanger's phenomenology. The latter's sense of autism as a radical loss of being-in-the-world is answered by contemporary advocacy and action toward greater awareness and accommodation of neurodiverse difference within a shared community.

The other thing that is juxtaposed in the accounts in this study are different positioned perspectives and associated partialities (or biases) in perception and evaluation. Such partiality is naturally most easily seen from a historical distance and is striking when one looks at the analytically informed accounts of the early twentieth century. Awareness of our equivalent partiality in the present takes more reflection. We have tried to emphasise the positionality of these different accounts in their context as they have been described. Sometimes, this partiality matters less. It takes only a few accounts of intense autistic worlds and elaborated sensitivity in autism to counter some of the more extreme deficit-based models which came from the behavioural turn and the downplaying of subjective experience, particularly within observation of children and a theoretical notion that autism was intrinsically related to language delay (something subsequently shown to be incorrect). But if we are to apply a more systematic and applicable phenomenology across the range of autism as currently apparent, then we are proposing the need for a more inclusive, systematic, and empirical approach—something that is outlined in the final section of the paper. This includes aspect of participatory science, new approaches to data collection and analysis (informed by citizen science models), and the development of new tools and technologies for non-speaking individuals to ensure that the range of autistic voices contributes to the development of the phenomenological research. This will not solve all the issues arising in this area, but it will be an approach that, if applied systematically over time, could overcome partiality in narratives and distil common representative elements of autistic phenomenology, foundational for a re-casting of the autism phenotype as it is currently framed.

## Data availability statement

The raw data supporting the conclusions of this article will be made available by the authors, without undue reservation.

## Ethics statement

Written informed consent was not obtained from the individual(s) for the publication of any potentially identifiable images or data included in this article because the case reports have been fully anonymised, including fundamental adaptation of names and case details so as to be non-identifiable, with quotes that are illustrative transliterations rather than verbatim records.

## Author contributions

JG: Conceptualisation, Investigation, Writing—original draft, Writing—review & editing. NS: Conceptualisation, Writing—original draft, Writing—review & editing.

## Footnotes

^1^We begin by establishing our own positionally and perspectives as authors. The first author (Green) is non-autistic. He has worked clinically as a child psychiatrist with autistic children, young people, and families for 35 years and as a research professor in autism developmental and intervention research. The second author (Shaughnessy) identifies as neurodivergent and has interdisciplinary expertise in creative research methods, autism, and mental health.

^2^Daniel Tammet (b. 1979) is the eldest of nine children from a working-class East London family. *Born on a Blue Day:A Memoir of Asperger's and an Extraordinary Mind* describes his atypical early development in ways that are characteristic of childhood autism, also documenting the onset of epilepsy (aged four) and the associated acquisition of synaesthesia. Educated in local mainstream schools and excelling academically (particularly in maths and languages), he started his career as a language teacher, launching an online language learning company in 2002. He was diagnosed in 2004 with high-functioning autistic savant syndrome and quickly came to public attention for his award-winning recitation of Pi, at the age of 25. *Blue Day* was published in 2005, followed by *Embracing the Wide Sky* (2009) (on neuroscience) and the essay collection, *Thinking in Numbers* (2014). Naoki Higashida (b. 1992) was educated in Japanese specialist schools after receiving an autism diagnosis at age five. He was supported to communicate using a Japanese alphabet grid and finger pointing which a helper transcribed. He wrote *The Reason I Jump* at age 13 which was published in Japan in 2007. It was translated into English and published in 2013 after being discovered by KA Yoshida, wife of the author David Mitchell who found the account pertinent to their autistic son. A sequel, *Fall Down 7 Times Get Up 8* (2017), documented Naoki's adolescent life. Whilst there is considerable controversy surrounding the authorship of these books due to criticism of the communication system and speculation about the writer's agency, *The Reason I Jump* (and the subsequent film documentary, 2020) is one of the few creative representations of young autistic people with complex communication needs. Anand Prahlad (b. 1954) was born on a plantation in Virginia, attending “the only black school in the area” (63). Although he did not speak until the age of four and was acutely aware of being different to his peers, his diagnosis of Asperger's was when he was 57. *The Secret Life of a Black Aspie* is a memoir of growing up undiagnosed and how the intersections of race and neurodivergence shaped his subjectivity. After an MA and PhD, he became an academic (teaching creative writing, folklore, and disability studies) alongside a professional career as a poet, non-fiction author, and musician. Katherine May (b.1977) was brought up in Kent and attended the University of Cambridge. She wrote *The Electricity of Every Living Thing* (2018) whilst going through the journey of diagnosis in her late 30s. The memoir is an example of a rapidly developing corpus of contemporary autobiographies by autistic women, whose experiences have until recently been under-represented. Following *Electricity*, May has further authored two acclaimed memoirs—*Wintering* (May, 2020) and *Enchantment* (May, 2023).

^3^Neurotypes is a term used by neurodiversity scholars referring to classes of “differently wired brains” (e.g., autistic, dyslexic, etc).

^4^An emphasis reduced as developmental science and epidemiology has progressed; with the disaggregation of language delay from autism nosology in DSM5.
